# Recommendations of the SFH (French Society of Haematology) for the diagnosis, treatment and follow-up of hairy cell leukaemia

**DOI:** 10.1007/s00277-014-2140-y

**Published:** 2014-07-05

**Authors:** Edouard Cornet, Alain Delmer, Pierre Feugier, Francine Garnache-Ottou, David Ghez, Véronique Leblond, Vincent Levy, Frédéric Maloisel, Daniel Re, Jean-Marc Zini, Xavier Troussard

**Affiliations:** 1 Haematology Laboratory, Caen University Hospital, 14033 Caen Cedex, France; 2 Department of Clinical Haematology, Reims University Hospital, 51092 Reims Cedex, France; 3 Haematology Division, Nancy University Hospital, 54035 Nancy Cedex, France; 4 EFS, BFC, 25020 Besançon Cedex, France; 5 Gustave Roussy, 94805 Villejuif Cedex, France; 6 Department of Clinical Haematology, Pitié Salpêtrière Hospital, 75651 Paris Cedex, France; 7 Haematology Oncology Thorax Division, Hôpital Avicenne, 93003 Bobigny Cedex, France; 8 Sainte Anne Clinic, 67085 Strasbourg Cedex, France; 9 Antibes Hospital, 06100 Nice Cedex, France; 10 Antoine Lacassagne Centre (Nice), 06100 Nice Cedex, France; 11 Saint Louis Hospital, 75010 Paris Cedex, France; 12 Haematology Laboratory, CHU de Caen, 14000 Caen, France

**Keywords:** Hairy cell leukaemia, Recommendations, Diagnosis, Treatment

## Abstract

Hairy cell leukaemia (HCL) is a rare haematological malignancy, with approximately 175 new incident cases in France. Diagnosis is based on a careful examination of the blood smear and immunophenotyping of the tumour cells, with a panel of four markers being used specifically to screen for hairy cells (CD11c, CD25, CD103 and CD123). In 2011, the V600E mutation of the *BRAF* gene in exon 15 was identified in HCL; being present in HCL, it is absent in the variant form of HCL (HCL-v) and in splenic red pulp lymphoma (SRPL), two entities related to HCL. The management of patients with HCL has changed in recent years. A poorer response to purine nucleoside analogues (PNAs) is observed in patients with more marked leukocytosis, bulky splenomegaly, an unmutated immunoglobulin variable heavy chain (*IgVH*) gene profile, use of *VH4–34* or with *TP53* mutations. We present the recommendations of a group of 11 experts belonging to a number of French hospitals. This group met in November 2013 to examine the criteria for managing patients with HCL. The ideas and proposals of the group are based on a critical analysis of the recommendations already published in the literature and on an analysis of the practices of clinical haematology departments with experience in managing these patients. The first-line treatment uses purine analogues: cladribine or pentostatin. The role of BRAF inhibitors, whether or not combined with MEK inhibitors, is discussed. The panel of French experts proposed recommendations to manage patients with HCL, which can be used in a daily practice.

## Background

The epidemiological data relating to hairy cell leukaemia (HCL) are limited. HCL is a rare haematological malignancy, representing 2 % of all leukaemia [1]. Between 1992 and 2000, a review of 12 American databases covering 14 % of the American population revealed a higher frequency of HCL among white Americans than among African Americans or Asians [2]. The data from the lower Normandy Regional Register of Haematological Malignancies indicate an incidence standardized to the world population of 0.29 ± 0.08 per 100,000. The incidence is higher in men (0.50 ± 0.15) than in women (0.12 ± 0.008) [3]. These data, extrapolated to France, suggest approximately 175 incident cases of HCL per year.

The median age of patients at diagnosis is 52 (24–81). The SEER (Surveillance, Epidemiology and End Results Program; http://seer.cancer.gov) study conducted in 3,776 patients shows an improvement in survival over the calendar periods analysed (before 1984, 1984–1990, 1991–1999 and 2000–2008) and a reduction in the risk of mortality of 6.5 % per year. The survival rate for HCL is lower among African Americans [4].

The causes of HCL remain unknown. The existence of familial forms suggests a genetic predisposition in some cases [1]. The role of certain environmental factors remains unclear; a risk associated with forage growing or exposure to organophosphate insecticides has been identified, as has a tobacco-associated protective effect [5]. In 2011, mutations were identified in five genes (*BRAF*, *CSMD3*, *SLC5A1*, *CNTN6* and *OR8J*) by means of whole-exome high-throughput sequencing performed on the (>90 %) CD19^+^ tumour cells from a patient with HCL and on the (>98 %) CD19^−^ mononuclear cells from the same patient at the time of remission [6]. The V600E mutation of the *BRAF* gene in exon 15 was confirmed by means of direct sequencing in 47 other patients with HCL. However, it was absent in 195 patients with another chronic B cell lymphoproliferative disorder, including patients with marginal zone lymphoma.

HCL may be associated with other haematological malignancies, particularly multiple myeloma (MM), large granular lymphocytic leukaemia (LGL) or chronic myeloid leukaemia (CML) [7].

## Diagnosis

The circumstances under which the disease is discovered are related to the consequences of bone marrow suppression, including severe or recurrent infections, to the detection of splenomegaly, whether symptomatic or not, or to the often fortuitous identification of hairy cells during a routine blood count.

In all cases, the diagnosis is based on a careful examination of the blood smear and immunophenotyping of the tumour cells.

### Identification of hairy cells in the blood smear

Hairy cells—large cells with abundant, poorly demarcated, grey to weakly and irregularly basophilic cytoplasm—display fine cytoplasmic projections distributed around the entire circumference of the cell. “Granular/lamellar” cytoplasmic inclusions with the appearance of slightly basophilic rods with a clear central area are detected occasionally. The nucleus-to-cytoplasm ratio is low, and the nucleus is often in an eccentric position. Oval or round, it can sometimes be kidney-shaped. The nuclear chromatin has a dispersed appearance and never coarse, and the nucleolus, which is not readily seen, is small and often solitary. Sometimes difficult to identify in a poor quality smear, the cells are found consistently in the blood smear, even if only in small numbers.

Monocytopenia is almost always present. It may be erroneously absent from the results yielded by automated haematology analysers, which regularly identify hairy cells as monocytes. There is no lymphocytosis. Neutropenia, anaemia that is often mildly macrocytic and thrombocytopenia of varying severity often complete the constellation of laboratory findings.

### Immunophenotype

Immunophenotyping can be performed on blood or bone marrow. Hairy cells must be looked for within a large cell gate (in the vicinity of the monocyte gate).

It comprises an analysis of the B cell lineage markers (CD19, CD20) combined with a panel of markers used specifically to screen for hairy cells (CD11c, CD25, CD103, CD123) and screening for an immunoglobulin light chain isotype restriction.

The four markers CD11c, CD25, CD103 and CD123 define the HCL score [8], which distinguishes HCL from other B cell haematological disorders associated with hairy cells, including the variant form of HCL (HCL-v), splenic marginal zone lymphoma (SMZL) and splenic red pulp lymphoma (SRPL). One point is given to each marker when it is expressed, and no point is given when it is not expressed. A score of 3 or 4 is observed in 98 % of cases of HCL, unlike with other B cell haematological disorders associated with hairy cells, where the score is usually 0 or 1. It is not compulsory to calculate this score, the co-expression of the markers CD11c, CD25 and CD103 representing a sufficient basis on which to diagnose HCL. If a negative result is obtained for one of these three markers, there is a need to assess the expression of CD123 and to calculate this score.

Although there is no need to calculate it for the diagnosis of HCL, the Royal Marsden Hospital (RMH) score [9] used in chronic lymphocytic leukaemia (CLL) is usually 0 or 1.

### Bone marrow examination

This is not obligatory, except in clinical trials.

Where diagnosis proves difficult or immunophenotyping is inconclusive and the number of hairy cells present is small, a bone marrow biopsy combined with bone marrow aspiration is desirable. Hairy cells, cells which are easily recognizable by their oval or kidney-shaped nuclei, their chromatin pattern, the clear “halo” which separates the nuclei and the abundant cytoplasm which is difficult to see, have a typical “fried egg” appearance.

Immunohistochemistry (IHC) is sometimes useful for detecting hairy cells in limited numbers—expression of the isoenzyme 5 of tartrate acid-resistant phosphatase (TRAP) in the form of non-specific but characteristic granular cytoplasm positivity, expression of CD20 and CD72 and the mutated BRAF protein [10].

### Cytogenetic and molecular analyses

Cytogenetic studies and *BRAF* V600E mutation testing [6,11] are not necessary for diagnosis. *BRAF* V600E mutation testing may be useful in cases of diagnostic uncertainty and in cases where therapeutic management is complex.

### Other investigations at diagnosis

Clinical evaluation must include the following:

- A patient history, looking for any familial history of haematological malignancy or autoimmune disorders;
- Identification of any systemic symptoms;
- Checking for signs of disease, especially checking for splenomegaly. The use of imaging can be helpful for this. Ultrasonography of the spleen can be used to measure the size of the spleen and to identify abdominal lymphadenopathy, which is uncommon.

Biological tests include the following:

- A complete blood count with differential including a reticulocyte count;
- Serum protein electrophoresis to look for a monoclonal component;
- Haemolysis screen in the presence of suspicion.

The following is desirable:

- Preservation of tumour material in blood and/or bone marrow before any treatment.

## Differential diagnosis

The variant form of HCL and SRPL constitutes forms which are difficult to diagnose with areas of overlap with HCL [12].

### Variant form of HCL

Despite being described as long ago as 1980 [13], the variant form of HCL (HCL-v) is a rare entity (10 % of all cases of HCL). The terminology is ambiguous, because it is a disorder distinct from HCL. It affects men, with a median age of 71 years (48–92 years) [14,15]. Monocytopenia is absent, and leukocytosis is >10 × 10^9^/L in more than 90 % of cases. Examination of the blood smear shows a proliferation of hairy cells (20–95 % of the lymphoid cells) of medium to large size with a large and clearly visible nucleolus and condensed chromatin. There is infiltration of the splenic red pulp. The HCL score is low (0, 1 or 2) with a strong expression of CD11c (87 %) and CD103 (60 %) contrasting with a weak, rare expression of CD123 (7 %) or CD25 (6 %) [15,16]. The *BRAF* V600E mutation is absent.

### Splenic red pulp lymphoma or SRPL

SRPL is rare [17] and was described recently. It affects men (sex ratio, 1.64) with a median age of 77 years (46–91). In three quarters of cases, lymphocytosis is present and examination of the blood smear shows the presence of a proliferation of hairy cells (median, 60 %; 26–91 % of the lymphoid cells) of small to medium size with, in the majority of cells, a small or invisible nucleolus. The cellular phenotype is very heterogeneous, quite close to HCL-v, with a strong expression of CD11c (97 %), a variable expression of CD103 (38 %) and little or no expression of CD123 (16 %) and CD25 (3 %). The blood karyotype may show anomalies such as 7q deletion. The immunoglobulin variable heavy chain (*IgVH*) gene profile is mutated in 80 % of cases, with over-represented use of VH3–26 and VH4–34. The BRAF V600E mutation is absent in SRPL.

## Required pretreatment evaluation

### Assessment of prognostic factors

Unless there is a therapeutic indication, there is no justification for carrying out any additional investigations for the assessment of prognostic factors. No prognostic factor will modify the choice of first-line treatment, which is based on purine nucleoside analogues (PNAs): cladribine or pentostatin.

Studies in patients treated with cladribine have resulted in the identification of factors, which may reduce the following responses to treatment:

- Leukocytosis >10 × 10^9^/L [18]
- Bulky spleen extending >10 cm below costal margin [18]
- Unmutated *IgVH* gene profile ≥98 % [18]
- Use of the *VH4–34* repertoire [19]
- Mutation of the *TP53* gene [18].

Other factors reduce event-free survival (EFS) [18] as follows:

- Bulky spleen extending >10 cm below costal margin
- Elevated levels of circulating hairy cells >10 × 10^9^/L
- Unmutated *IgVH* profile.

*TP53* mutation testing and analysis of the IgVH gene repertoire to test for VH4–34 positivity must be performed in the event of a relapse, before the second line of treatment, on previously frozen material or fresh material if possible.

### Pretreatment assessment

Parameters considered necessary for a complete pretreatment evaluation may differ depending on whether or not the patient is treated in a clinical protocol.

The following are essential pretreatment tests (clinical trials and general practice):

- Physical examination including the determination of the performance status as defined by the Eastern Cooperative Oncology Group (ECOG) and the identification of comorbidities and potential sites of infection.
- Serum chemistry including creatinine with calculation of creatinine clearance (Modification of Diet in Renal Disease (MDRD) Study), haemolysis screen (direct antiglobulin test, haptoglobin, unconjugated bilirubin and lactic dehydrogenase) and liver function tests (transaminases) with hepatitis B and C serology (risk of reactivation after immunosuppressive therapy).
- Preservation of cells and serum in a tumour bank is recommended.
- Chest radiograph and abdominal and pelvic imaging.

The following are additional pretreatment tests that may be performed in clinical trials:

- Blood and bone marrow karyotypes analysis.
- *BRAF* V600E mutation testing.
- Mutational status of *IgVH* gene and analysis of the VH gene usage.
- Mutational status of *TP53* gene.

## Treatment

### Indications for treatment

Criteria for initiating treatment may vary depending on whether or not the patient is treated in a clinical trial. In general practice, treatment is indicated in the presence of at least one of the signs of active disease as follows:

1. Symptomatic splenomegaly
2. Cytopenia (involving at least one cell type)
  - Haemoglobin <10 g/dL
  - Platelets <100 × 10^9^/L
  - Neutrophils <1 × 10^9^/L
3. Recurrent or severe infections

In the absence of treatment, clinical monitoring and monitoring of the blood count are necessary every 3 months for the first year and then every 6 months.

### Treatment strategy

Inclusion in a prospective study protocol is recommended. In general practice, the recommendations are as follows.

#### First-line treatment

The first-line treatment is based on PNAs [20–22]: cladribine or pentostatin. The different dosing regimens for the two medicinal products are listed in Table 1.

**Table 1:** Different dosing regimens

Dosing regimen for cladribine (2-chlorodeoxyadenosine (2-CDA))
Authorized dosages
0.14 mg/kg/day SC for 5 days
0.1 mg/kg/day as a continuous IV infusion for 7 days
Other dosages (off-label)
0.14 mg/kg/day as an IV infusion over 2 h for 5 days
0.14 mg/kg/day IV once weekly for 6 weeks
0.14 mg/kg/day SC once weekly for 5 weeks
The dosage must be repeated in the absence of CR at 6 months
Dosing regimen for pentostatin in HCL (2′-deoxycoformycin (2′-DCF))
4 mg/m^2^ every 2 weeks until a maximal response is achieved, plus one or two additional injections
Determine the creatinine clearance before each administration and avoid the medicinal product in the presence of clearance <60 mL/min
Dosing regimen for rituximab (4–8 doses in total)
375 mg/m^2^ IV once weekly administered simultaneously or sequentially with the purine analogue in patients without CR after a single treatment with pentostatin or cladribine
Interferon alpha (IFN-α)
3 × 10^6^ U SC daily until a maximal response is achieved and continuation at the same dose three times weekly. In very cytopenic patients, start on a dose of 3 × 10^6^ U 3 times weekly
Splenectomy
Indicated in cases of bulky splenomegaly (>10 cm below the costal margin) and in cases of moderate bone marrow involvement, after immunization programme

There are no data in the literature proving the superiority of one drug over the other. The choice between pentostatin and cladribine is based essentially on the presence or absence of renal impairment, the severity of the cytopenias and the dosing regimens of the products. Cladribine may be used intravenously (IV) or subcutaneously (SC), unlike pentostatin, which is used only via the IV route. The SC form offers ease of administration and the option of treatment on an outpatient basis, unlike cladribine IV.

In the presence of renal impairment (plasma creatinine clearance (MDRD) <60 mL/min), pentostatin is contraindicated owing to limited experience, unlike cladribine.

#### Special cases

In cases of severe neutropenia (neutrophil count <0.2 × 10^9^/L) and/or uncontrolled active infection, the treatment of choice is interferon via the SC route at a dose of 3 million units × 3 per week followed by a purine analogue once the neutropenia has been corrected. Another option, although not validated in this indication, is the use of rituximab on its own at a dose of 375 mg/m^2^ IV for 4 weeks in the place of interferon.

In cases of active haemolytic anaemia, consideration might be given to rituximab.

In cases of pregnancy, the only treatment that can be used is interferon via the SC route at a dose of 3 million units × 3 per week.

In cases of two co-existing haematological disorders or a concomitant haematological disorder, the management approach should be discussed at a multidisciplinary meeting.

#### Relapses

Only symptomatic relapses require treatment.

##### First relapse

In cases of late relapse (>60 months), PNAs may be used again. No recommendations exist regarding whether or not the PNAs should be changed.

In cases of intermediate relapse (24–60 months), the second-line treatment also relies on PNAs, whether or not a different PNA is used and whether or not it is combined with rituximab, administered concomitantly or sequentially (non-validated treatment regimens) [23,24].

In cases of early relapse (<24 months), the diagnosis of HCL must be confirmed and *BRAF* V600E mutation testing must be performed.

1. If the diagnosis of HCL is confirmed, the management approach will depend on the presence or absence of a *TP53* gene mutation and VH4–34 use.
  - In the absence of the *TP53* gene mutation, with a VH repertoire other than VH4–34, the use of a PNA combined with rituximab is recommended.
  - In cases of *TP53* gene mutation [25] and/or use of VH4–34, the different treatment options are as follows:
    Immunotoxins, but they are not available in France [26,27].
    BRAF inhibitors either alone or in combination with MEK inhibitors. No treatment regimen is defined at present [28–31]. The use of these treatments must take into account the toxicity of these drugs, including their cutaneous effects [30,32,33].
    Bendamustine and rituximab in combination [34].
    If access to innovative compounds is impossible, a combination of treatment with PNA and rituximab is recommended.
2. If the diagnosis of HCL is not confirmed and in the absence of a *BRAF* V600E mutation, an analysis of the *IgVH* repertoire to test for *VH4–34* use must be performed. Treatment must be considered on a case-by-case basis.

##### Subsequent relapses

Some options used before the era of PNAs should not be ruled out, including splenectomy and interferon.

### Prevention of infectious complications

The prevention of infectious complications occurring during the course of HCL treatment or in the post-treatment period is of prime importance.

In the prevention of *Pneumocystis jiroveci*-induced infections with Bactrim® until recovery of T cell numbers to CD4 > 200/mm^3^, there is an increased risk of allergic skin reactions during concomitant use of Bactrim® with purine analogues. This risk can be reduced by starting Bactrim® 1 week after the purine analogue. In cases of allergy to Bactrim® or cytopenias potentially linked to Bactrim®, Pentacarinat® or atovaquone (Wellvone®) aerosols may be used.

Prevention of zoster infections with aciclovir or valaciclovir should also be started 1 week after PNAs. The optimal duration of prophylaxis has not been determined.

Growth factors should be considered on a case-by-case basis.

No data in the literature provide a basis for either recommending or not recommending the irradiation of labile blood products after treatment with purine analogues.

## Response criteria and evaluation

The following definitive response should be assessed:

- For pentostatin, at the end of treatment, after 8 to 10 cycles
- For cladribine, 3 to 6 months after the end of treatment.

Complete remission (CR) is defined as follows:

- Recovery of blood counts with haemoglobin >12 g/dL, platelets >150 × 10^9^/L, neutrophils >1.5 × 10^9^/L and absence of circulating hairy cells
- Regression of splenomegaly
- Absence of hairy cells on the bone marrow biopsy specimen, including IHC analyses (CD20, CD72).

In the absence of a bone marrow biopsy specimen, a very good response (VGR) is defined as recovery of the blood counts (haemoglobin >12 g/dL, platelets >150 × 10^9^/L, neutrophils >1.5 × 10^9^/L and absence of circulating hairy cells) and regression of splenomegaly.

A partial response (PR) is defined as a poorer quality haematological response, with persistence of splenomegaly, cytopenias or presence of circulating hairy cells on blood smear analysis.

In cases of PR, the treatment approach depends on the desired objective.

If *quality of life* is a priority (elderly subject, comorbidities), regular haematological monitoring (clinical examination and complete blood count) every 6 months should be recommended.

If *response quality* is sought (young subject), achieving complete remission (CR) is desirable. CR should be defined as a negative bone marrow biopsy, including IHC, and ideally minimal residual disease (MRD) which is undetectable in the blood and/or bone marrow by means of flow cytometry. The impact of detectable MRD on the risks of relapse and on prognosis remains unknown, however [35,36]. In the absence of CR, a new treatment, either with the same PNA or with a combination of cladribine and rituximab, is recommended.
